# A new insight into the immobilization mechanism of Zn on biochar: the role of anions dissolved from ash

**DOI:** 10.1038/srep33630

**Published:** 2016-09-19

**Authors:** Tingting Qian, Yujun Wang, Tingting Fan, Guodong Fang, Dongmei Zhou

**Affiliations:** 1 Key Laboratory of Soil Environment and Pollution Remediation, Institute of Soil Science, Chinese Academy of Sciences, Nanjing 210008, China; 2 University of Chinese Academy of Sciences, Beijing 100049, China

## Abstract

Biochar is considered to be a promising material for heavy metal immobilization in soil. However, the immobilization mechanisms of Zn^2+^ on biochars derived from many common waste biomasses are not completely understood. Herein, biochars (denoted as PN350, PN550, WS350, and WS550) derived from pine needle (PN) and wheat straw (WS) were prepared at two pyrolysis temperatures (350 °C and 550 °C). The immobilization behaviors and mechanisms of Zn^2+^ on these biochars were systematically investigated. The results show that compared with biochars produced at low temperature, biochars produced at high temperature contained higher amounts of ash and exhibited much higher sorption capacities of Zn^2+^. By using Zn K-edge EXAFS spectroscopy, we find that the formation of various Zn precipitates/minerals, which was caused by the release of OH^−^, CO_3_^2−^, and Si species from biochar, was the immobilization mechanism of Zn^2+^ on PN and WS biochars. Hydrozincite and Zn(OH)_2_ were the main species formed on PN350, PN550, and WS350; while on WS550, besides hydrozincite, a large fraction of hemimorphite was formed. The occurrence of hydrozincite and hemimorphite on biochar during Zn^2+^ immobilization is firstly reported in our study, which provides a new insight into the immobilization mechanism of Zn^2+^ on biochar.

Zinc (Zn) is an essential nutrient for living organisms^1^, however, an excess supply of Zn can lead to toxic effect on plants^2^,^3^,^4^ and microorganisms^5^,^6^ in soil. Due to anthropogenic activities (such as industrial activities, sludge application, waste water irrigation, etc.^3^), Zn can be easily released into soil environment^7^, which could pose a threat to the health of ecosystems^8^ and even to human beings^9^,^10^. As free Zn ions are the main species for Zn toxicity^1^,^5^, the immobilization of free Zn ions by some materials would reduce its bioavailability and toxicity in soil. Thus, materials which are effective in Zn immobilization should be developed.

As an emerging carbonaceous material, biochar has been extensively studied especially in the field of soil remediation^11^,^12^,^13^,^14^,^15^,^16^,^17^. The carbonaceous residue and entrained minerals (ash) are two main components of biochar^18^, they perform different functions in soil remediation. The carbonaceous residue can adsorb and retain water^19^ and some organic pollutants^20^,^21^,^22^,^23^; the ash can provide nutrient elements and improve soil pH^24^. In terms of heavy metal immobilization, both of the two components could be involved in the process. Plenty of studies have reported the immobilization behavior of Zn on biochars derived from different biomass (such as hardwood^25^,^26^, corn straw^25^, sugarcane straw^27^, dairy manure^28^, and meat and bonemeal^29^), and some of them have proposed the immobilization mechanisms which implied more important role of ash on biochar on Zn immobilization^27^,^28^,^30^. Xu *et al.* studied the sorption behavior of Zn on biochar derived from dairy manure, and found that large amounts of PO_4_^3−^ and CO_3_^2−^ were released from biochar and reacted with Zn^2+^ to form Zn phosphate and Zn carbonate^28^. With the help of P K-edge XANES spectroscopy, Wegner *et al.* found that hopeite (Zn_3_(PO_4_)·2H_2_O) or a similar Zn-P phase were formed, when biochar was used to immobilize Zn in the sewage field soils^30^. In addition to forming Zn precipitates, the formation of Zn complexes on biochar surface also led to Zn immobilization^29^. By using the method of extended X-ray absorption fine structure (EXAFS) spectroscopy, Betts *et al.*^29^ found that Zn bound to phosphate groups in meat and bonemeal biochar in a monodentate inner-sphere surface complex.

Feedstock types and pyrolysis temperature are two main factors influencing biochar property^18^. As a wide variety of biomasses can be used for biochar production, previous studies on Zn^2+^ immobilization behavior of limited type of biochars are far from enough. To explore the immobilization mechanisms of Zn^2+^ on different biochars and provide more basic data for biochar application, studies which are focused on biochars produced by other waste biomasses need to be done. Although many studies have mentioned the effect of pyrolysis temperature on biochar property^20^,^31^,^32^,^33^,^34^, few studies systematically investigated the effect of pyrolysis temperature on immobilization behavior of Zn^2+^ on biochar and elucidated the internal connection between pyrolysis temperature, biochar property, and immobilization behavior of Zn^2+^ on biochar. In this study, biochars produced by pine needle (PN) and wheat straw (WS) were chosen for investigation, to our knowledge, there is no published work concentrated on Zn^2+^ immobilization behavior of biochars derived from these biomasses. Two temperatures were chosen for biochar preparation, and the Zn^2+^ immobilization behaviors of biochars produced by different temperature were compared through several sorption experiments (i.e. sorption kinetics, sorption isotherms, and the effect of pH). To reveal the importance of the ash on biochars on Zn immobilization, the ash was removed from biochars, and the Zn^2+^ immobilization behaviors of de-ashed biochars were compared with those of the raw biochars. To explore the immobilization mechanisms, the methods of Zn K-edge EXAFS spectroscopy and X-ray Powder Diffraction (XRD) were used for biochar characterization. Through this study, the Zn species immobilized by PN and WS biochars will be identified, the immobilization mechanisms and the relation between pyrolysis temperature and immobilization behaviors of Zn^2+^ on studied biochars will be clear.

## Results and Discussion

### Characterization of PN and WS biochars

The contents of the common elements (i.e. K, Ca, Mg, Al, Fe, Zn, and P) in biochars were determined and listed in Table 1. The contents of most studied elements in biochars produced at 550 °C were higher than those in biochars produced at 350 °C, which suggested that the ash contents of biochars produced at 550 °C were higher than those of biochars produced at 350 °C. The high Ca contents in PN biochars and high K contents in WS biochars implied that, in addition to immobilizing heavy metal, PN and WS biochars could also supply large amounts of nutrient elements to the soil. When raw biochars were washed by HCl/HF solution, the contents of the elements substantially dropped, this means that the factor of ash which may influence the sorption of Zn can be excluded in de-ashed biochars. The pHs of raw biochars and the pH_PZC_ of de-ashed biochars were also listed in Table 1.

To characterize the functional groups on different biochars, FTIR and XPS analysis were conducted on these biochars. The results were shown in Supplementary Fig. S1 and Supplementary Fig. S2 and Table S1.

### Sorption kinetics and isotherms and the effect of initial pH on sorption behavior of Zn^2+^ on biochars

**Figure 1:**
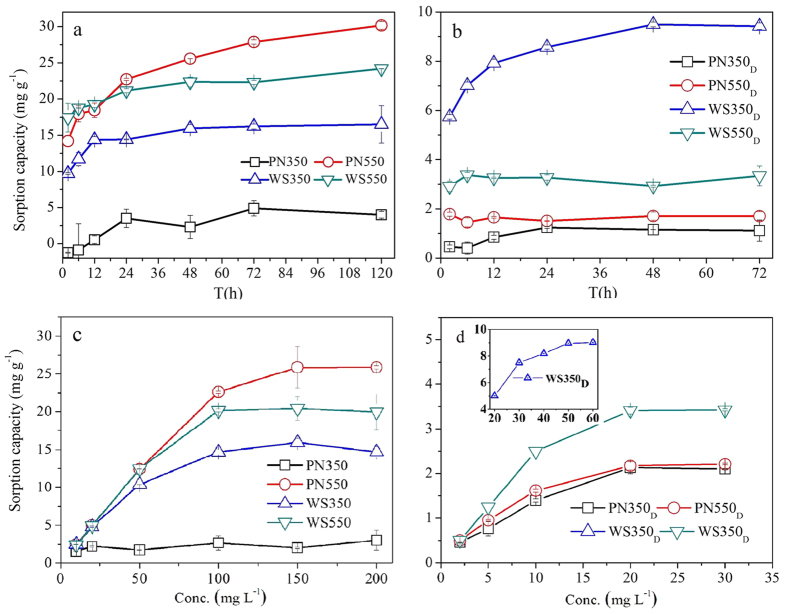
Kinetics and isotherms of Zn^2+^ sorption on different biochars. (**a**) Kinetics of Zn^2+^ sorption on raw biochars (the initial concentration of Zn was 150 mg L^−1^; the solution pHs were ranged from 6.5 to 7.5 (see Fig. S3) without adjustment; (**b**) kinetics of Zn^2+^ sorption on de-ashed biochars (the initial concentration of Zn was 50 mg L^−1^; the solution pHs were controlled to 7.0); (**c**) isotherms of Zn^2+^ sorption on raw biochars (the solution pHs were not adjusted); (**d**) isotherms of Zn^2+^ sorption on de-ashed biochars (the solution pHs were controlled to 7.0). Error bars represent ± SE (SE is the standard error of estimate). The experiments were conducted in duplicate (n = 2).

To study the sorption behaviors of Zn^2+^ on different biochars, a series of sorption experiments were conducted. Figure 1 shows the kinetics and isotherms of Zn^2+^ sorption on different biochars. From Fig. 1a we find that sorption reactions between Zn^2+^ and most biochars can reach equilibrium at 48 h, the sorption capacities of Zn^2+^ on biochars produced at 550 °C were higher than those of Zn^2+^ on biochars produced at 350 °C. Figure 1c shows that the maximum sorption capacities of Zn^2+^ on raw biochars decreased in the following order: PN550 (25.9 mg g^−1^) > WS550 (20.4 mg g^−1^) > WS350 (16.0 mg g^−1^) > PN350 (3.0 mg g^−1^). It seems that biochars with higher ash contents were more effective on Zn immobilization. From Fig. 1c,d we find that, the maximum sorption capacities of Zn^2+^ on de-ashed biochars were lower than those of Zn^2+^ on their corresponding raw biochars. Especially for biochars produced at 550 °C, the maximum sorption capacities of Zn^2+^ on biochars decreased by more than 80% when the ash was removed. These results suggested the important role of ash on Zn immobilization by biochar. Among de-ashed biochars, WS350_D_ had the highest sorption capacity (9.4 mg g^−1^), which may be due to the more oxygen-containing functional groups WS350_D_ had compared with other biochars. Kinetics experiments show that sorption reactions between Zn^2+^ and most biochars can reach equilibrium at 48 h, thus the equilibrium time for the following experiments was set to be 48 h. As PN550, WS350, and WS550 had considerable sorption capacities of Zn^2+^, several kinetics models (pseudo-first and pseudo-second-order models^35^, Elovich model^36^,^37^, and intraparticle diffusion model^38^,^39^) and isotherms models (Langmuir, Frendlich, and Langmuir-Frendlich models^40^) were performed on kinetics and isotherms data of these biochars, the fitting results were shown in Supplementary Tables S2 and S3.

The effect of initial pH on sorption capacities of Zn^2+^ on biochars were shown in Supplementary Fig. S4. As can be seen from Fig. S4, the sorption capacities of PN550, WS350, and WS550 decreased gradually with the decrease of initial pH. Unlike the sorption behaviors of raw biochars, sharp decreases occurred on the sorption capacities of de-ashed biochars, when the initial pH decreased from 9 to 7. This may be caused by the absence of buffer effect of ash. The low sorption capacity of Zn^2+^ on PN350 under the whole pH range could be due to the low ash content.

### XRD analysis

**Figure 2:**
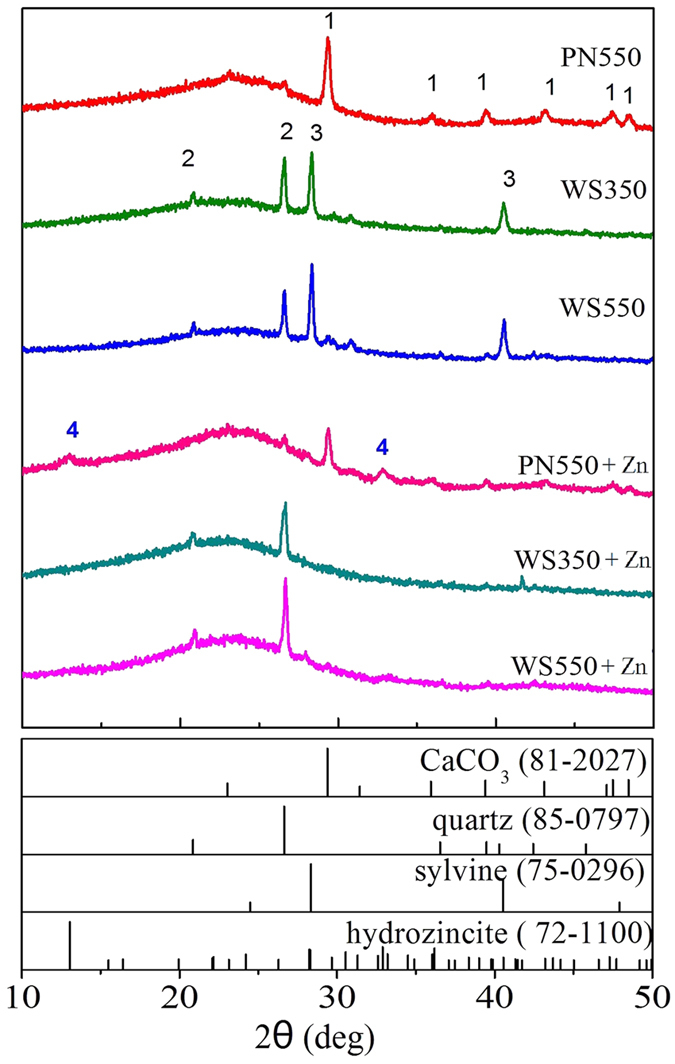
The XRD spectra of raw biochars and biochars loaded with Zn^2+^. (The species are 1. CaCO_3_ (81–2027), 2. quartz (SiO_2_, 85–0797), 3. sylvine (KCl, 75–0296), 4. hydrozincite (Zn_5_(CO_3_)_2_(OH)_6_, 72–1100)).

The sorption experiments showed that some Zn precipitates/minerals may be formed on biochar, thus XRD analysis were performed on these biochars. As PN350 had a relatively low sorption capacity of Zn^2+^, we just focused on other three biochars (PN550, WS350, and WS550) and biochars loaded with Zn (denoted as PN550 + Zn, WS350 + Zn, and WS550 + Zn). Figure 2 shows the XRD spectra of the samples. The main mineral in PN550 was CaCO_3_, the peaks of quartz in PN550 were not obvious. The peak intensities of CaCO_3_ and quartz show that there could be large amount of CO_3_^2−^ and low amount of Si contained in PN550. In WS350 and WS550, two minerals can be detected, they were quartz and sylvine. Compared with PN550, WS350 and WS550 contained larger amount of Si species.

Compared with the spectra of PN550, two more peaks appeared in the spectra of PN550 + Zn, which may belong to hydrozincite. Thus the formation of hydrozincite could be one of the mechanisms for Zn immobilization on PN550. In WS350 + Zn and WS550 + Zn, as sylvine is soluble, only quartz was maintained on WS350 and WS550. Although WS350 and WS550 had considerable sorption capacities of Zn^2+^, none of the Zn species could be detected from the two samples by XRD analysis. This may be caused by the poor crystallinity of Zn species formed on WS350 and WS550. As the characterization way of XRD has the requirement on sample crystallinity, the method of XAFS was applied in Zn species identification.

### EXAFS analysis

**Figure 3:**
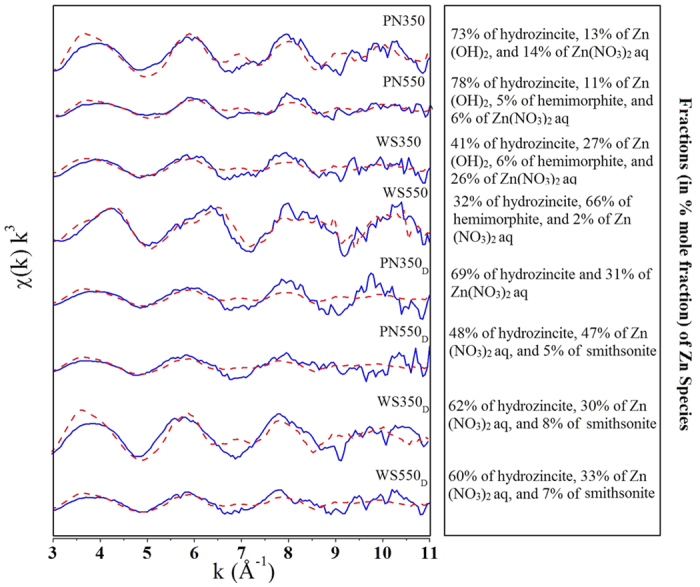
χ(k) k^3^-spectra of the Zn species on different biochars (solid line) and fractions of Zn species (in % mole fraction) determined by linear combination fitting. Deshed line represents the fitted line.

The k^3^-weighted χ(k) functions of raw biochars and de-ashed biochars are shown in Fig. 3. To obtain the types and fractions of Zn species on these biochars, Principal Component Analysis (PCA) accompanied with Target Transformation (TT) and Linear Combination Fitting (LCF) were performed on χ(k) k^3^-spectra ranging from 3–11 Å^−1^ for all the samples. PCA was used to determine the number of primary components which may be present in the spectra of biochars; TT was used to evaluate which species may be contained in these biochars; and LCF was applied to quantitatively analyze the Zn species on biochars. (The details on PCA, TT and LCF were shown in S1 in SI).

The result of PCA shows that five principal components may be present in the spectra of biochars. After TT by using the *χ*(k) k^3^-spectra of a series of standard Zn compounds (Supplementary Fig. S5), we found six Zn species (i.e. Zn(NO_3_)_2_ aqueous (Zn(NO_3_)_2_ aq), Zn(OH)_2_, willemite (Zn_2_SiO_4_), smithsonite (ZnCO_3_), hemimorphite (Zn_4_(H_2_O)(Si_2_O_7_)(OH)_2_), and hydrozincite (Zn_5_(CO_3_)_2_(OH)_6_)) may be contained in these biochars. The number of Zn species obtained by TT was higher than that obtained by PCA, this is due to the similarity between the spectra of some standard species^41^,^42^,^43^. The PCA results suggested that the Zn precipitates/minerals could take up a large fraction of Zn species in biochar.

The LCF was subsequently performed on the samples using the Zn species selected by TT. The main components and their fractions in the samples were obtained and shown in Fig. 3 and Supplementary Table S6. From the fitting results (Fig. 3), we find that Zn precipitates/minerals (i.e. hydrozincite, Zn(OH)_2_, and hemimorphite) accounted for a large share of Zn species (>70%) in raw biochars, which indicated the indispensable role of ash in biochar on Zn immobilization. High proportions of hydrozincite (>40%) and Zn(OH)_2_ (>10%) were observed in most of the raw biochars. This result indicated that CO_3_^2−^ and OH^−^ were two common ions for Zn immobilization. It is noteworthy that, unlike other raw biochars, WS550 contained large fraction of hemimorphite (66%). This was probably caused by the large amount of dissolved Si species released from WS550, which implied the crucial role of dissolved Si species in Zn immobilization. The species Zn(NO_3_)_2_ aq represents the Zn adsorbed on oxygen-containing functional groups (i.e. carboxyl and hydroxyl) on biochar. In PN350 and WS350, the fractions of Zn(NO_3_)_2_ aq were respectively 14% and 26%; while in PN550 and WS550, the two values dropped to 6% and 2%. These changes were due to the increase in ash contents and loss of oxygen-containing functional groups in biochars with pyrolysis temperature. In de-ashed biochars, only two main Zn species, hydrozincite and Zn(NO_3_)_2_ aq, existed in de-ashed biochars. Although hydrozincite is Zn mineral, the formation of small amounts of hydrozincite in de-ashed biochars may be caused by the dissolution of CO_2_ in the air. The pH_PZC_ of de-ashed biochars were 4.3–5.5 (Table 1), thus when the solution pHs were controlled to 7, the de-ashed biochars could easily adsorb few amount of Zn^2+^.

### The Ions Involved in Zn Immobilization

According to the analysis of XRD and EXAFS, we have identified the main Zn species formed on biochars. To examine these results, the pHs of the samples withdrawn at 2 h and the concentrations of CO_3_^2−^ and Si species in the samples withdrawn at 120 h in kinetics experiments were determined. For comparison, these determinations were also conducted on their corresponding blank samples (0.1 g of biochar + 25 mL of water). The concentrations of PO_4_^3−^, Ca^2+^, and Mg^2+^ were also determined. The samples in kinetics experiments are denoted as B + Zn, and the blank samples are denoted as B.

As shown in Table 2, the pHs of B + Zn at the reaction time of 2 h reached 7.0, which were lower than those of B. This indicated that plenty of OH^−^ were consumed during Zn immobilization. For PN350 and WS350, the concentrations of CO_3_^2−^ in B were respectively 0.3 and 1.1 mmol L^−1^; while the concentrations of CO_3_^2−^ in B + Zn could not be detected (ND) indicating the consumption of CO_3_^2−^ during Zn immobilization. As Ca^2+^ which released from biochars could also react with CO_3_^2−^, the amount of CO_3_^2−^ which would react with Zn to form hydrozincite would be underestimated from these results. The released Si species could also influence the determination of CO_3_^2−^, as they could buffer H^+^ in the solution^44^, which lead to the overestimated concentrations of CO_3_^2−^. Thus the concentrations of CO_3_^2−^ in different samples cannot be used to calculate the real amounts of consumed CO_3_^2−^ in Zn immobilization, they were only for reference. For WS350 and WS550, the Si concentrations in B were respectively 0.9 and 2.0 mmol L^−1^ (the initial concentration of Zn^2+^ was 2.3 mmol L^−1^). Once reacted with Zn^2+^, the concentrations of Si decreased drastically. From the considerable amounts of released and consumed Si species, we assumed that some Si species in WS biochars may play an important role in Zn immobilization. Fig. S6 shows the ATR-FTIR spectra of the raw biochar filtrates. From Figs S6a and S6b we cannot see any band present in the spectra, while in Figs S6c and S6d there is a broad band in the region 1150–1050 cm^−1^ in each of the spectrum, which is due to the asymmetric Si–O–Si stretching vibration of long chain polymers^45^. The ATR-FTIR result suggested that the Si species released from biochar are Si-containing polymers with different chain length. The release and consumption of OH^−^, CO_3_^2−^ and Si species confirmed the results of XRD and EXAFS.

The difference on the concentrations of PO_4_^3−^ between B and B + Zn implied the formation of Zn_3_(PO_4_)_2_ on biochars, however the concentrations of PO_4_^3−^ in B were no more than 0.06 mmol L^−1^, thus the precipitation role of PO_4_^3−^ in the studied biochars can be ignored. The concentrations of Ca^2+^ and Mg^2+^ in B + Zn were much higher than those of Ca^2+^ in B; expect for the condition of PN350, the concentrations of Mg^2+^ in B + Zn were also higher than those of Mg^2+^ in B. This may be caused by the competition of Zn^2+^ for released OH^−^, CO_3_^2−^, and some sorption sites. When raw biochar was added into water, plenty of ions such as Ca^2+^, Mg^2+^, OH^−^, and CO_3_^2−^ were released from biochar to bulk solution. With the gradual increase in concentrations of Ca^2+^, Mg^2+^, and anions such as OH^−^, CO_3_^2−^ in the solution close to biochar surface, some of these ions would be over-saturated, Ca, Mg-precipitates would form and deposit on the surface of biochar. In addition to forming precipitates, a portion of Ca^2+^ and Mg^2+^ could also be captured by oxygen-containing functional groups of biochar. Thus, in raw biochar-water systems, the newly released Ca^2+^ and Mg^2+^ can be immobilized rapidly. When Zn^2+^ was present in the solution, Zn^2+^ would diffuse to the area close to biochar surface, they could also react with the newly released anions (i.e. OH^−^ and CO_3_^2−^) and occupy the sorption sites (i.e. oxygen-containing functional groups of biochar). This process would decrease the concentrations of OH^−^, CO_3_^2−^, and PO_4_^3−^ and sorption sites of Ca^2+^ and Mg^2+^, which further resulted in the increased amounts of released Ca^2+^ and Mg^2+^ in raw biochar-Zn solution systems (B + Zn). The low sorption capacity of Zn^2+^ on PN350 and inhomogeneous distribution of Mg on biochar may lead to the lower concentration of Mg^2+^ in B(PN350) + Zn, when compared with those of Mg^2+^ in B(PN350) + Zn.

### The Possible Evolution Processes of OH^−^, CO_3_^2−^, and Si Species on Biochars

To reveal the immobilization mechanism of Zn on biochars, we first need to know the formation rule of OH^−^, CO_3_^2−^, and Si Species. Although few studies can describe the clear evolution processes of ash in biochar, we may obtain the general evolution processes based on the pyrolysis rule of biomass.

In plants, some alkali and alkaline earth metal (AAEM), e.g. K, Ca, and Mg, are associated with organic molecules^46^. During pyrolysis, with the decomposition of the organic matters and reconstruction of the remaining parts, biomass transformed into char (not the ultimate biochar), and AAEMs were then bonded to the char in the form of C–O–Met^+^ or C–O–Met^2+^–O–C (Met^+^ represents K^+^, and Met^2+^ represents Ca^2+^ and Mg^2+^)^47^. Once the bonds between AAEMs and char were attacked by free radicals which were formed in pyrolysis, elemental AAEMs were formed^47^,^48^,^49^,^50^. As these species are not stable, they would be easily oxidized to form metal oxides and react with H_2_O, which was released from biomass during pyrolysis, to form AAEM hydroxides (see Equation 1, M represents elemental AAEMs, such as K, Ca, or Mg). These AAEM hydroxides could be the source of released OH^−^ in the solution. The decomposition of cellulose and lignin^18^,^51^ lead to the generation of CO_2_. CO_2_ may react with H_2_O and newly formed AAEM hydroxides to form various carbonates (see Equations 2 and 3). Thus, in addition to the carbonates contained in the raw biomass, the newly formed carbonates during pyrolysis could also be the source of CO_3_^2−^ in the solution. The Si species are much more stable than other inorganic species in biomass^18^,^52^. It is reported that the main forms of Si in wheat biomass are amorphous silica and silicate esters^53^,^54^, the forms of these Si species would change when the water in biomass evaporated and the organic matters decomposed during pyrolysis, some of the Si species would transform into quartz (see Fig. 2) and dehydroxylated silicates^18^, some of them would transformed into alkali silicates (such as K_2_SiO_3_)^52^. Without the protection of organic matters, the exposed Si species can be easily released to the solution. Among the Si species formed on biochar, alkali silicates are soluble compounds, the solutions of which contain a wide variety of polysilicate ions^55^ (consist with the ATR-FTIR results of raw biochar filtrates). Thus alkali silicates are probably the Si species released into water or Zn solution.


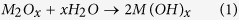



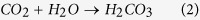



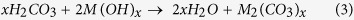


From the proposed evolution processes of the anions, we find that the formation of these ions are closely related to the decomposition of biomass, and the higher degree of decomposition (higher pyrolysis temperature) will lead to the higher amounts of OH^−^, CO_3_^2−^, and Si species presented on biochar surface. Some previous studies have also found this phenomenon. Yuan *et al.* reported that the pHs and carbonate contents of biochar derived from different straws increased with pyrolysis temperature^56^; Xiao *et al.* found that the dissolved Si species released from biochar prepared by rice straw increased^33^ when the temperature increased from 250 °C to 700 °C.

### The Immobilization Mechanism of Zn on PN and WS Biochars

**Figure 4:**
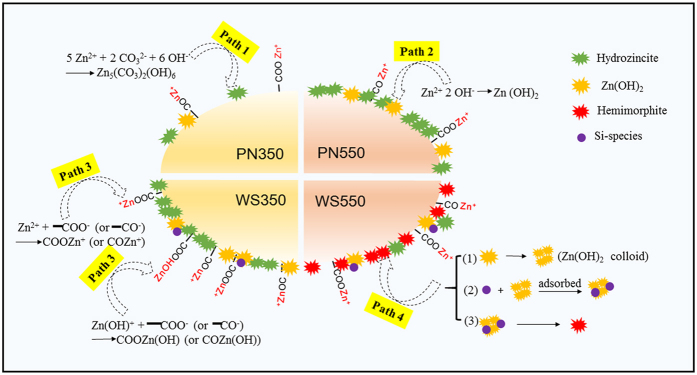
The formation process of the main Zn species on different biochars.

The formation of various ashes on biochars is the premise for Zn immobilization; and the formation of different Zn species is the immobilization mechanism of Zn on PN and WS biochars. The formation processes of the main Zn species (the fractions of which are higher than 10%) on different biochars are proposed and described as follows (Fig. 4).

For PN350 and WS350, once biochars were added into Zn solution, the OH^−^ and CO_3_^2−^, which were formed on biochar during pyrolysis^56^, tend to release into bulk solution. As OH^−^ and CO_3_^2−^ were released from biochar, at the early stage of the diffusion the concentrations of OH^−^ and CO_3_^2−^ in the solution close to biochar surface were much higher than those of bulk solution. Under this condition, when Zn^2+^ diffused to biochar surface, these ions were easily over-saturated and form hydrozincite and Zn(OH)_2_ (Fig. 4, Paths 1 and 2), and finally deposited on biochar surface. For biochars produced at low temperature (i.e. 350 °C), there existed a large portion of oxygen-containing functional groups (i.e. carboxyl and hydroxyl)^24^. When they presented in the solution with high pH, these functional groups will be deprotonated and adsorb Zn^2+^. During the process of Zn immobilization, the solution pHs were not controlled, they decreased from >9 to 7 with reaction time. From the calculation of Visual MINTEQ 3.1 (Supplementary Fig. S7), we find that two Zn species (i.e. Zn^2+^ and Zn(OH)^+^) could be involved in the reaction, and the proportion of Zn^2+^ was much higher than that of Zn(OH)^+^. The reactions were shown in Fig. 4, Path 3. Although the sorption mechanisms of PN350 and WS350 are the same, the sorption capacity of Zn on PN350 are much lower than that on WS350, which may be due to the lower amounts of OH^−^, CO_3_^2−^ and oxygen-containing functional groups formed on PN350 when compared with those formed on WS350. This phenomenon implied that different feedstocks have different degree of decomposition under the same pyrolysis temperature, which would lead to different sorption performances of biochars.

For PN550 and WS550, with the volatilization of organic matters and completion of thermal decompositions, the ash content increased, which made the fraction of Zn precipitates/minerals close to 100%. Compared with PN350, PN550 contained much more CO_3_^2−^ and OH^−^, thus the sorption capacity of Zn on PN550 was higher than that of PN350. For WS550, a large fraction of hemimorphite was formed. This was due to a considerable amount of released Si species. The formation of hemimorphite on WS550 can be divided into three steps^57^ (Fig. 4, Path 4): (1) the formation of colloid of Zn(OH)_2_ under alkaline condition, (2) the adsorption of dissolved silicates on Zn(OH)_2_ colloid and co-precipitation of silicate and Zn(OH)_2_, and (3) the reconstruction of the co-precipitates structure and formation of amorphous hemimorphite. Due to the complexity of the sorption system and short reaction time, the well-crystallized hemimorphite was unlikely to form. Thus, it cannot be detected by the method of XRD. Although there were also some Si species released from WS350, only a small portion of hemimorphite was formed. This may be caused by the low amounts of OH^−^ and Si species released from WS350 biochar.

The formation of different Zn species on biochars confirmed heterogeneous property of these materials which resulted in the co-existence of various sorption mechanisms during Zn retention by biochars. The consumption of OH^−^, CO_3_^2−^, or the sorption sites (oxygen-containing functional groups) on biochars by Zn^2+^ to form different Zn species led to the release of Ca^2+^ and Mg^2+^. When the studied raw biochars were added into the acid solutions, the ions OH^−^ and CO_3_^2−^ in biochar were consumed and even some oxygen-containing functional groups were protonated. This was against the formation of different Zn species, which could lead to the decrease in the sorption capacities of Zn on biochars.

## Conclusion

The ash in PN and WS biochars played important role on Zn immobilization. The higher pyrolysis temperature on the feedstock leads to the higher sorption capacity of Zn^2+^ and the larger fraction of Zn precipitates/minerals on biochar. Hydrozincite and Zn(OH)_2_ were the main species formed on PN350, PN550, and WS350; while on WS550, besides hydrozincite, a large fraction of hemimorphite was formed. The formation of OH^−^, CO_3_^2−^, and Si species on biochars and the release of these species played predominant role on Zn immobilization.

## Materials and Methods

### Preparation of Raw Biochars and De-ashed Biochars

Biochars derived from PN and WS were prepared in a patented biochar reactor (NO. ZL2009 2 0232191.9) under oxygen-limited conditions. According to the decomposition rule of the feedstocks obtained from thermal analysis, 350 °C and 550 °C were selected as the pyrolysis temperatures (See Supplementary S2). To remove the ash on biochars, HCl/HF (1.0 M, v/v 1:1) solution was used to wash the raw biochars for several times. The details for biochar preparation were shown in Supplementary S2. The biochar sample derived from PN (WS) at 350 °C and 550 °C were denoted as PN350 (WS350) and PN550 (WS550), respectively. The de-ashed biochar were denoted as PN350_D_, PN550_D_, WS350_D_, and WS550_D_.

### Sorption Kinetics and Sorption Isotherms

One hundred milligram of raw biochars or de-ashed biochars were added into 25 mL of Zn(NO_3_)_2_ solutions in 50 mL vials. These vials were shaken at 200 rpm at 25 °C. For raw biochars, the Zn^2+^ concentration in Zn(NO_3_)_2_ solution was 150 mg L^−1^ and the pH of the solution was not adjusted. For the sorption experiments of de-ashed biochars, the Zn^2+^ concentration was 50 mg L^−1^ and the pH of the solution was adjusted to 7 buffered by 5 mM MOPs. The samples were withdrawn at appropriate time intervals, then the mixture was filtered with a 0.45 μm membrane, and the Zn^2+^ concentrations of the filtrates were determined. The sorption capacity of biochar in each time interval was obtained by Supplementary Equation S1. The concentrations of CO_3_^2−^, Si species, PO_4_^3−^, Ca^2+^, and Mg^2+^ were also determined in the samples withdrawn at 120 h, then concentrations of these five species in the filtrates of blank samples (0.1 g of biochar + 25 mL of water) at 120 h were determined for comparison. The concentrations of Zn^2+^, Ca^2+^ and Mg^2+^ were determined using Atomic Absorption Spectrometer (AAS, Hitachi-Z2000, High-Technologies Corporation, Japan); the concentrations of CO_3_^2−^ were determined using titration method^44^; the concentrations of Si species were determined using Inductively Coupled Plasma − Atomic Emission Spectrometer (ICP-AES, OPTIMA 8000, PerkinElmer, USA); and the concentrations of PO_4_^3−^ were determined using molybdate-ascorbic acid method. Two parallel samples were applied in sorption samples. Sorption isotherms were performed the same way as sorption kinetics except for the initial concentrations of Zn^2+^ applied (see Supplementary S6).

### Effect of Initial pH

Fifty milligram of raw biochars or de-ashed biochars were added into 23 mL of deionized (DI) water in 50 mL vials. These vials were shaken at 200 rpm at 25 C for 3 day equilibrium. Then the pHs of the mixtures in these vials were adjusted to 2–10. Certain amount of Zn(NO_3_)_2_ stock solution was dripped into each vial to make the initial concentration of Zn^2+^ to be 150 mg L^−1^ for raw biochar and 50 mg L^−1^ for de-ashed biochar. DI water was then added into the vial to make the solution volume up to 25 mL. The following procedure was the same as that of isotherms experiments and the sorption capacity of biochar was obtained by Supplementary Equation S6.

### Characterization of Feedstocks and Biochars

The thermal properties of PN and WS were obtained using a Thermogravimertic Analyzer (TGA, Pyris 1 TGA, PerkinElmer, USA) at 20 °C/min up to the final temperature of 700 °C under a flow of N_2_. The functional groups of biochar and the Si species in the filtrates of raw biochar were analyzed using Fourier Transform Infrared Spectroscopy (FTIR, Nicolet FTIR IS10, Thermo Scientific, USA). The methods of sample preparation and data analysis are shown in Supplementary S8. The surface of biochar was analyzed with X-ray Photoelectron Spectrometer (XPS, PHI-5000 Versaprobe, ULVAC-PHI, Japan) using an Al Kα excitation radiation. The pHs of raw biochars and pHs of point of zero charge (pH_PZC_) of de-ashed biochars were obtained by the pH drift method in Supplementary S9 and S10. The contents of K, Ca, Mg, Al, Fe, Zn, and P in biochars were analyzed after the microwave digestion of biochars with HNO_3_-HF-H_2_O_2_ (U.S. EPA 3502)^58^. The concentrations of K, Al, and Fe in the digestion solution were determined with ICP-AES. The digestion experiments were conducted in duplicate for each biochar.

### Characterization of Zn Species on Biochars

The minerals in biochars were analyzed by X-ray Powder Diffraction (XRD). The tests were performed on a Rigaku, Ultima IV Diffractmeter (Rigaku Corporation, Japan), and Cu Kα radiation generated at 40 kV/40 mA, data were collected in the range (2θ) from 0° to 60° with the scan step of 0.02°. The minerals in the samples were identified using the XRD data analysis software (MDI JADE 6.5) and its corresponding powder diffraction file (PDF) database. The Zn species on biochars were analyzed by the method of extended X-ray Absorption Fine Structure (EXAFS) spectroscopy in fluorescence modes. Zn K-edge (9659 eV) measurements were carried out at beamline BL14W at the Shanghai Synchrotron Radiation Facility Center (SSRF). Principal Component Analysis (PCA) accompanied with Linear Combination Fitting (LCF) were used for species identification and quantification. The details of EXAFS spectra collection and data processing were shown in Supplementary S11.

## Additional Information

**How to cite this article**: Qian, T. *et al.* A new insight into the immobilization mechanism of Zn on biochar: the role of anions dissolved from ash. *Sci. Rep.* **6**, 33630; doi: 10.1038/srep33630 (2016).

## Supplementary Material

Supplementary Information

## Figures and Tables

**Table 1:** The contents of some common elements (mg g^−1^), and the pH and pH_PZC_ of biochars.

Biochar	K	Ca	Mg	Al	Fe	Zn	P	pH and pH_PZC_*
PN350	9.8 ± 0.6	15.7 ± 0.3	3.0 ± 0.2	1.4 ± 0.0	0.9 ± 0.0	0.076 ± 0.001	1.8 ± 0.0	7.9 ± 0.1
PN550	13.6 ± 0.0	23.7 ± 0.7	4.6 ± 0.2	2.1 ± 0.0	1.3 ± 0.0	0.106 ± 0.003	2.7 ± 0.0	9.6 ± 0.0
WS350	31.8 ± 0.4	9.7 ± 0.2	2.6 ± 0.0	1.8 ± 0.0	1.7 ± 0.0	0.061 ± 0.009	1.3 ± 0.0	8.2 ± 0.0
WS550	38.8 ± 0.6	12.3 ± 0.7	3.8 ± 0.1	2.3 ± 0.1	2.2 ± 0.0	0.060 ± 0.001	1.7 ± 0.0	9.7 ± 0.0
PN350_D_	0.29 ± 0.02	5.0 ± 0.5	1.8 ± 0.0	0.46 ± 0.00	0.25 ± 0.01	0.056 ± 0.002	0.12 ± 0.00	4.3 ± 0.1
PN550_D_	0.24 ± 0.01	4.7 ± 0.1	4.0 ± 0.1	0.91 ± 0.02	0.28 ± 0.00	0.083 ± 0.003	1.20 ± 0.02	5.4 ± 0.1
WS350_D_	0.02 ± 0.00	1.0 ± 0.1	0.7 ± 0.0	0.13 ± 0.05	0.21 ± 0.01	0.016 ± 0.003	0.05 ± 0.00	4.4 ± 0.0
WS550_D_	0.06 ± 0.00	3.3 ± 0.1	2.2 ± 0.0	0.12 ± 0.01	0.31 ± 0.01	0.029 ± 0.002	0.22 ± 0.00	5.5 ± 0.1

^*^The dissolution of the ash in raw biochar could consume a large amount of H^+^, which makes the pH_PZC_ data of raw biochars unreliable, thus only the pH_PZC_ of de-ashed biochars has been tested.

**Table 2:** pH and Concentrations of Species (mmol L^−1^) Involved in Zn Immobilization in the Samples.

Biochars	pH*		CO_3_^2−^		Si species		PO_4_^3−^		Ca^2+^		Mg^2+^	
	B	B + Zn	B	B + Zn	B	B + Zn	B	B + Zn	B	B + Zn	B	B + Zn
PN350	8.3 ± 0.1	6.7 ± 0.1	ND	ND	0.01 ± 0.00	0.001 ± 0.000	0.03 ± 0.00	ND	0.08 ± 0.00	0.8 ± 0.2	0.07 ± 0.00	0.05 ± 0.00
PN550	10.0 ± 0.0	7.0 ± 0.1	0.3 ± 0.3	ND	0.05 ± 0.00	0.02 ± 0.00	0.04 ± 0.00	ND	0.10 ± 0.01	1.4 ± 0.0	0.06 ± 0.00	0.09 ± 0.00
WS350	9.5 ± 0.0	7.1 ± 0.0	ND	ND	0.89 ± 0.02	0.09 ± 0.00	0.06 ± 0.00	ND	0.08 ± 0.00	0.5 ± 0.1	0.09 ± 0.00	0.18 ± 0.02
WS550	10.4 ± 0.0	7.2 ± 0.1	1.1 ± 0.0	ND	1.95 ± 0.05	0.23 ± 0.00	0.06 ± 0.00	ND	0.11 ± 0.00	0.5 ± 0.1	0.08 ± 0.00	0.17 ± 0.02

^*^As some ions released from biochar may consume released OH^−^ in biochar + water mixture as reaction time increases, the pHs of B and B + Zn were determined at the reaction time of 2 h.
