# Supplementary material for: A new insight into the immobilization mechanism of Zn on biochar: the role of anions dissolved from ash
Source: Sci Rep. 2016 Sep 19;6:33630. doi: 10.1038/srep33630 (PMC5027529; doi:10.1038/srep33630)
Supplement: Supplementary Information [file srep33630-s1.pdf]

Supplementary information

A new insight into the immobilization mechanism of Zn on biochar: the role of anions dissolved from ash

Tingting Qian[1], Yujun Wang[1], Tingting Fan[1, 2], Guodong Fang[1], and Dongmei Zhou[1,*]

[1] Key Laboratory of Soil Environment and Pollution Remediation, Institute of Soil Science, Chinese Academy of Sciences, Nanjing 210008, China

[2] University of Chinese Academy of Sciences, Beijing 100049, China

*Corresponding author, Tel: 0086-25-86881180, Fax: 0086-25-86881000, E-mail: dmzhou@issas.ac.cn (Dongmei Zhou)

Supplementary Information consists of 27 pages, including this one.

There are 6 Tables and 8 Figures.

S1. PCA, TT, and LCF

PCA was used to determine the number of primary components which may be present in the spectra of biochars. The parameters, such as eigenvalues, the indicator values (IND) number, etc. were used as the criterion to evaluate how many components are included in studied spectra.[1-5] However, in our study, eigenvalues and IND failed to give reasonable results (Table S4), thus visual examination of the samples and model spectra were used to evaluate the primary components.[1] Finally, the first five components were chosen for target transformation (TT). A series of Zn compounds (Table S5) were used as standard species (The χ(k) k^3-spectra of these standard species are shown in Figure S5). The quality of TT can be evaluated by the SPOIL value (Table S5). The lower the value is, the greater the likelihood the standard species is contained in the samples.[6] In this study, the standard species with SPOIL values lower than 3 were considered to be present in the samples. As was shown in Table S5, there are six Zn species (i.e. $Zn(NO_3)_2$ aqueous ($Zn(NO_3)_2$ aq), $Zn(OH)_2$, willemite (Zn_2SiO_4), smithsonite ($ZnCO_3$), hemimorphite ($Zn_4(H_2O)(Si_2O_7)(OH)_2$), and hydrozincite ($Zn_5(CO_3)_2(OH)_6$)) whose SPOIL values are lower than 3.

The LCF was subsequently performed on the samples using the Zn species selected by TT. For raw biochars, all the six Zn species were used for the fitting. As five principal components were statistically identified by PCA, only the combinations containing less than five components were chosen to model the samples. While for de-ashed biochars, willemite and hemimorphite cannot form as most of the ash was removed from biochar. During the sorption experiment, the pHs of de-ashed biochars

and Zn solution mixtures were controlled at 7.0. According to the calculation of Visual MINTEQ 3.1, $Zn(OH)_2$ would not be produced, however, the Zn species hydrozincite and smithsonite would form. Thus when LCF was performed on the spectra of de-ashed biochars, only $Zn(NO_3)_2$ aq, smithsonite, and hydrozincite were chosen for the fitting. The smallest R-factor and the sum of all fractions close to 100% are two criterions to identify the most appropriate combination. By these criterions, the main components and their fractions in the samples were obtained.

S2. Pyrolysis temperatures

In order to gain large differences on properties of biochars derived from the same feedstock, TG and DTG analysis were conducted on PN and WS. Figure S8 shows the TG and DTG curves of PN and WS. The decomposition of PN and WS can be roughly divided into two stages: the evaporation of water ranging from 40 to 130 ^{o}C and the decomposition of cellulose, hemicellulose, and lignin[7] ranging from 200 ^{o}C to 500 ^{o}C for PN and 250 ^{o}C to 400 ^{o}C for WS. The maximum decomposition rates of PN and WS were respectively at 380 and 340 ^{o}C, which indicative of the decomposition of cellulose. As lignin is composed of aromatic rings with different branches, it has a wide decomposition temperature range between 160 to 900 ^{o}C.[8, 9] When the temperature was over 500 ^{o}C, the carbonaceous matters transformed from the feedstock continuously decomposed, but at a very low rates. According to the decomposition rule of the feedstock obtained from thermal analysis, 350 ^{o}C and 550 ^{o}C were selected as the pyrolysis temperatures. At 350 ^{o}C, the samples were only partially carbonized, and some ash may be formed and exposed to the environment, simultaneously plenty of

oxygen-containing functional groups were present on biochar surface.[10, 11] While at 550 ^{o}C, most of the organic matters had been evolved[12] and the sample was well carbonized.[10, 11] With the volatilization of a large amount of gaseous products, the oxygen-containing functional groups substantially decreased, and more ash was formed and exposed to the environment. The difference in degree of decomposition of the same feedstock led to the difference in their physicochemical properties, which would further influence their sorption behavior.

S3. Preparation of Raw and De-ashed Biochars

The pre-dried pine needle (PN) and wheat straw (WS) were cut into pieces, then ground into powder and passed through a 60-mesh (150 μm) sieve. The prepared feedstocks were dried and stored in a sealed plastic bag until use.

Biochars derived from PN and WS were prepared in a patented biochar reactor (NO. ZL2009 2 0232191.9) under oxygen-limited conditions. The prepared feedstock was firstly put into a crucible and compacted with a pestle, then moved into the biochar reactor. The feedstock was heated from ambient temperature to target temperature (i.e. 350 ^{o}C or 550 ^{o}C, the target temperature was selected according to the results of TG analysis.) with an average heating rate of 15 $^{o}C\ min^{-1}$. After being remained at the target temperature for 2 h the reactor was shut down. When cooling to the ambient temperature, the freshly prepared biochar was stored in a sealed plastic bag at the temperature of 4 ^{o}C.

To remove the ash in biochar, 15 g biochar was firstly washed by 500 mL 1 M HCl solution for four times, then by 500 mL HCl/HF (1.0 M, v/v 1:1) solution for four times,

each wash process lasted for 10 h. Finally, the sample was washed with deionized water for several times to remove the soluble salts. The de-ashed biochar was dried in an oven at 80 ^oC for 12 h. After cooling, the de-ashed biochar was stored as the same way as freshly prepared biochar.

S4. Calculation of Sorption Capacity of Zn^{2+} on Biochar

The sorption capacity of Zn^{2+} on biochar in each time interval (q_t, mg g^{-1}) was obtained by Equation S1:

$$q_t = \frac{(C_0 - C_t)V}{m} \quad \text{(S1)}$$

where C_0 and C_t (mg L^{-1}) are the Zn^{2+} concentration at time 0 and time t; V (L) is the volume of $Zn(NO_3)_2$ solution and m (g) is the amount of biochar added into the solution.

S5. Sorption Kinetics Models

Several mathematical models were used for kinetics data fitting. These models are pseudo-first (Equation S2) and pseudo-second-order models[13] (Equation S3), Elovich model[14, 15](Equation S4), and intraparticle diffusion model[16] (Equation S5) and the common equations of these models are shown as follows:

$$\ln(q_e - q_t) = \ln q_e - k_1 t \quad \text{(S2)}$$

$$\frac{t}{q_t} = \frac{1}{k_2 q_e^2} + \frac{t}{q_e} \quad \text{(S3)}$$

$$q_t = (1/\beta)ln(\alpha\beta) + (1/\beta)ln\, t \quad \text{(S4)}$$

$$q_t = k_d t^{\frac{1}{2}} \quad \text{(S5)}$$

where q_t and q_e(mg g^{-1}) are the amount of Zn^{2+} adsorbed at time t (h) and equilibrium, respectively; k_1 (h^{-1}) and k_2 (g mg^{-1} h^{-1}) are rate constants of the pseudo-first-order and the pseudo-second-order, respectively; α (mg g^{-1} h^{-1}) is the initial Zn^{2+} sorption rate, β

((mg g^{-1} h^{-1})$^{-1}$) is Zn desorption constant; k_d is constant. q_e and k_1 (in Equation S2) are calculated from the intercept and slope of the plot of ln (q_e–q_t) versus t, and q_e and k_2 (in Equation S3) are calculated from the intercept and slope of the plot of t/q_t versus t, respectively.

S6. Sorption Isotherms

The procedure of sorption isotherms experiments is the same to that of sorption kinetics experiments, except for the initial concentrations of Zn^{2+} (i.e. 10, 20, 50, 100, 150, and 200 mg L^{-1} for raw biochars; 20, 30, 40, 50, and 60 mg L^{-1} for $WS350_D$; 2, 5, 10, 20, and 30 mg L^{-1} for other de-ashed biochars) and the equilibrium time (i.e. 48 h according to the kinetics experiments). The sorption capacity of biochar (q_e, mg g^{-1}) was obtained by Equation S6:

$$q_e = \frac{(C_0 - C_e)V}{m} \qquad \text{(S6)}$$

where C_0 and C_e (mg L^{-1}) are the initial concentration of Zn^{2+} and the concentration of Zn^{2+} after 48 h.

S7. Sorption Isotherms Models

Langmuir, Frendlich, and Langmuir-Frendlich models were used for data fitting.

$$q_e = \frac{K_l Q C_e}{1 + K_l C_e} \qquad \text{(S7)}$$

$$q_e = K_f C_e^{\,n} \qquad \text{(S8)}$$

$$q_e = \frac{K_{lf} Q C_e^{\,n}}{1 + K_{lf} C_e^{\,n}} \qquad \text{(S9)}$$

Where q_e (mg g^{-1}), Q (mg g^{-1}), and C_e (mg L^{-1}) represent the amount of Zn^{2+} adsorbed at equilibrium, the maximum sorption capacity of Zn^{2+} on biochar, and the equilibrium

concentration of Zn^{2+}. K_l (L mg^{-1}), K_f (mg$^{(1-n)}$ L^n g^{-1}), and K_{lf} (L^n mg^{-n}) are langmuir isotherm constant, Frendlich isotherm constant, Langmuir-Frendlich isotherm constant, n is isotherm exponent. In this study, non-linear forms of the models and non-linear regression were applied to the sorption data, and a non-linear chi-square (χ^2) test were used to evaluate the fit of the model to the sorption data. The smaller the value of χ^2 is, the more similar the data from model and the data from experiments.[17]

$$\chi^2 = \sum \frac{(q_{e,exp} - q_{e,m})^2}{q_{e,m}} \quad \text{(S10)}$$

Where $q_{e,exp}$ (mg g^{-1}) is the amount of Zn^{2+} adsorbed at equilibrium in experiment; $q_{e,exp}$ (mg g^{-1}) is the calculated data from model.

S8. FTIR and ATR-FTIR measurement

To characterize the functional groups of biochar, a small amount of biochar was mixed with KBr powder, and then compressed into a pellet for determination. The solid sample (KBr pellet) were analyzed in transmission mode. To characterize the Si species in the filtrates of raw biochars, 0.2 g of biochar was added into 20 mL water, the sample was shaken at 200 rpm and at 25 ^{o}C. After 24 h, the sample was withdrawn and filtered with a 0.45 μm membrane. The filtrates of each biochar and water were conducted on ATR-FTIR analysis. The final ATR-FTIR spectra of each biochar was obtained by subtracting the spectra of water from the spectra of the filtrate of each biochar.

S9. The pHs of Raw Biochars

One hundred milligram of raw biochar was added into 25 mL deionized water in 50 mL vials and shaken for 24 h. The finial pH was determined and recorded as the pH of biochar. All analyses were conducted in duplicate.

S10. The pHs of Point of Zero Charge (pH_{PZC}) of De-ashed Biochars

A solution of 0.01 M KNO_3 which was boiled for the removal of dissolved CO_2 was prepared. The initial pHs of the solutions were adjusted by 0.1 M NaOH and 0.1 M HNO_3 solutions, and the pH range was from 2 to 8 according to the properties of the de-ashed biochars. Twenty five milligram of de-ashed biochars were added into 5 mL of pH-adjusted KNO_3 solutions in 10 mL vials. These vials were shaken for 48 h at 200 rpm at 25 ^{o}C. The final pHs of the solution were tested. The pH_{PZC} was the pH which did not change after 48 h equilibrium.[18] All analyses were conducted in duplicate.

S11. EXAFS Measurement

Zn K-edge (9659 eV) measurements were carried out at beamline BL14W at the Shanghai Synchrotron Radiation Facility Center (SSRF). The beam energy was 3.5 GeV and the beam current ranged from 150 to 210 mA. The spectra of biochars were collected at room temperatures in fluorescence modes with a double-crystal Si(111) monochromator.

The spectrum of Zn metal foil was used for energy calibration. The zero crossing of the second derivative of each sample spectrum was chosen as edge energy (E_0). Except for the values of Rbkg (set as 1.4, the default value is 1.0), the parameters for background removal and data normalization were default values of Athena. The normalized χ(E) data were transformed to k space (χ(k)) weighted by multiplying k^3. The k^3-weighted χ(k) functions (χ(k) k^3) were then Fourier transformed to derive radial structure functions (RSF).

The identification and quantification of Zn species on biochars were accomplished

by using the methods of Principal Component Analysis (PCA), Target Transformation (TT) and Linear Combination Fitting (LCF). The software SIXpack was used for PCA and TT, and Athena was used for LCF. The three methods were performed on $\chi(k)$ k^3-spectra ranging from 3~11 $Å^{-1}$ for all the samples. The spectra of Zn standards were kindly offered by Khaokaew et al. [19] and Cai et al.[20]

Table S1 The XPS analysis of different biochars

Raw biochars	B.E. (e.V)	species	PPA[1] (%)	De-ashed biochars	B.E. (e.V)	species	PPA (%)
PN350	284.4	C-C, Ar-C	52.6	$PN350_D$	284.6	C-C, Ar-C	64.6
	285.3	C defects	19.7		285.5	C defects	19.7
	286.3	C-O	12.2		286.5	C-O	14.5
	288.3	O-C=O	15.5		288.8	O-C=O	1.2
PN550	284.6	C-C, Ar-C	48.1	$PN550_D$	284.8	C-C, Ar-C	50.5
	285.2	C defects	27.6		285.4	C defects	26.3
	286.4	C-O	22.4		286.6	C-O	18.4
	289.3	π-π* transition	1.9		289.1	π-π* transition	4.8
WS350	284.3	C-C, Ar-C	22.5	$WS350_D$	284.7	C-C, Ar-C	33.5
	284.8	C defect	47.8		285.6	C defects	35.7
	285.9	C-O	24.3		286.7	C-O	23.5
	287.5	C=O	5.4		288.7	O-C=O	7.3
WS550	284.7	C-C, Ar-C	77.2	$WS550_D$	284.7	C-C, Ar-C	78.2
	286.3	C-O	21.7		286.3	C-O	17.9
	289.4	π-π* transition	1.1		288.8	π-π* transition	3.9

1. The proportion of peak area.

Table S2 The fitting parameters of kinetic models				
**Biochars**	**Fitting parameters**			
	Pseudo-first-order			
	k_1	q_e	$q_{e, exp}$	R^2
PN550	0.026±0.001	15.5±0.8	30.2±0.5	0.987
WS350	0.044±0.005	5.7±1.0	16.5±2.6	0.941
WS550	0.019±0.004	6.0±0.8	24.2±0.0	0.843
	Pseudo-second-order			
	k_2	q_e	$q_{e, exp}$	R^2
PN550	0.005±0.001	31.1±1.0	30.2±0.5	0.994
WS350	0.025±0.004	16.8±0.1	16.5±2.6	0.999
WS550	0.018±0.008	24.2±0.5	24.2±0.0	0.997
	Elovich			
	β	α		R^2
PN550	0.25±0.02	59.0±14.4		0.967
WS350	0.59±0.06	382.0±133.2		0.932
WS550	0.62±0.05	3.52×104±9846		0.957
	Intraparticle diffusion			
	k_d			R^2
PN550	1.66±0.14			0.958
WS350	0.66±0.15			0.975
WS550	0.67±0.07			0.941

As PN550, WS350, and WS550 had considerable sorption capacities of Zn^{2+}, several mathematical models including pseudo-first and pseudo-second-order models,[13] Elovich model,[14, 15] and intraparticle diffusion model[16, 21] were performed on kinetics data of these biochars, the fitting results were shown in Table S2. From the fitting parameters and coefficients of correlation (R^2), we can see that pseudo-second-order model had the best fit for all the tested biochars with R^2 all higher than 0.99, which suggested that chemical sorption or chemisorption may be the rate-limiting step for the sorption of Zn^{2+} on PN550, WS350, and WS550.[13]

**Table S3 The fitting parameters of isotherms models**					
Fitting parameters					
Langmuir					
	K_l (L mg^{-1})	Q (mg g^{-1})		R^2	χ^2
PN350	-	-		-	-
PN550	0.008±0.003	43.9±7.4		0.968	3.503
WS350	0.019±0.006	20.5±2.2		0.954	1.505
WS550	0.014±0.001	20.5±4.6		0.940	3.954
Frendlich					
	K_f (mg$^{(1-n)}$ L^n g^{-1})	n		R^2	χ^2
PN350	-	-		-	-
PN550	1.11±0.63	0.61±0.12		0.922	8.630
WS350	1.52±0.80	0.45±0.11		0.859	4.560
WS550	1.49±0.94	0.52±0.13		0.860	9.530
Langmuir-Frendlich					
	K_{lf} (L^n mg^{-n})	Q	n	R^2	χ^2
PN350	-	-	-	-	-
PN550	0.0014±0.0014	25.9	1.63±0.30	**0.986**	**1.490**
WS350	0.0034±0.0035	14.7	1.61±0.33	**0.977**	**0.742**
WS550	0.0012±0.0016	20.0	1.83±0.40	**0.975**	**0.159**

By comparing the values of R^2 and χ^2, we found that the sorption data could be fitted well with Langmuir-Frendlich model, which suggested the heterogeneity of biochars and the complexity of the sorption reaction between Zn^{2+} and biochars.[22]

Table S4 Parameters of the principal component analysis				
Component	eigenvalue	Variance	Cumulative Variance	IND
Component 1	104.8	0.427	0.427	0.467
Component 2	40.1	0.163	0.590	0.514
Component 3	30.4	0.124	0.714	0.601
Component 4	21.2	0.086	0.801	0.814
Component 5	18.2	0.073	0.874	1.197
Component 6	14.7	0.059	0.934	2.030
Component 7	9.4	0.038	0.973	6.540
Component 8	6.5	0.026	1.0	NA

**Table S5 Spoil values for target transformation**		
Zn species	Chemical formula	Spoil
HA-Zn	Zn coordinate with humic acid	3.4
$Zn(NO_3)_2$ aqueous	Hydrate Zn	**1.9**
Hopeite	$Zn_3(PO_4)_2$	3.3
$Zn(OH)_2$	$Zn(OH)_2$	**2.5**
Zincite	ZnO	4.4
Willemite	Zn_2SiO_4	**2.1**
Sphalerite	ZnS	4.4
Smithsonite	$ZnCO_3$	**2.3**
Hemimorphite	$Zn_4(H_2O)(Si_2O_7)(OH)_2$	**1.3**
Hydrozincite	$Zn_5(CO_3)_2(OH)_6$	**2.5**
Gahnite	$ZnAl_2O_4$	4.5
Franklinite	(Zn, Mn) Fe_2O_4	3.1

**Table S6. Fractions (in % mole fraction) of Zn species determined by LCF**								
Sample	Willemite	Smithsonite	Hydrozincite	$Zn(OH)_2$	Hemimorphite	$Zn(NO_3)_2$ aq	sum	R-factor
PN350			73	13		14	100	0.46
PN550			78	11	5	6	100	0.38
WS350			41	27	6	26	100	0.47
WS550			34		70	2	106	0.27
$PN350_D$			69			31	100	0.65
$PN550_D$		5	48			47	100	0.71
$WS350_D$		8	62			30	100	0.54
$WS550_D$		7	60			33	100	0.55

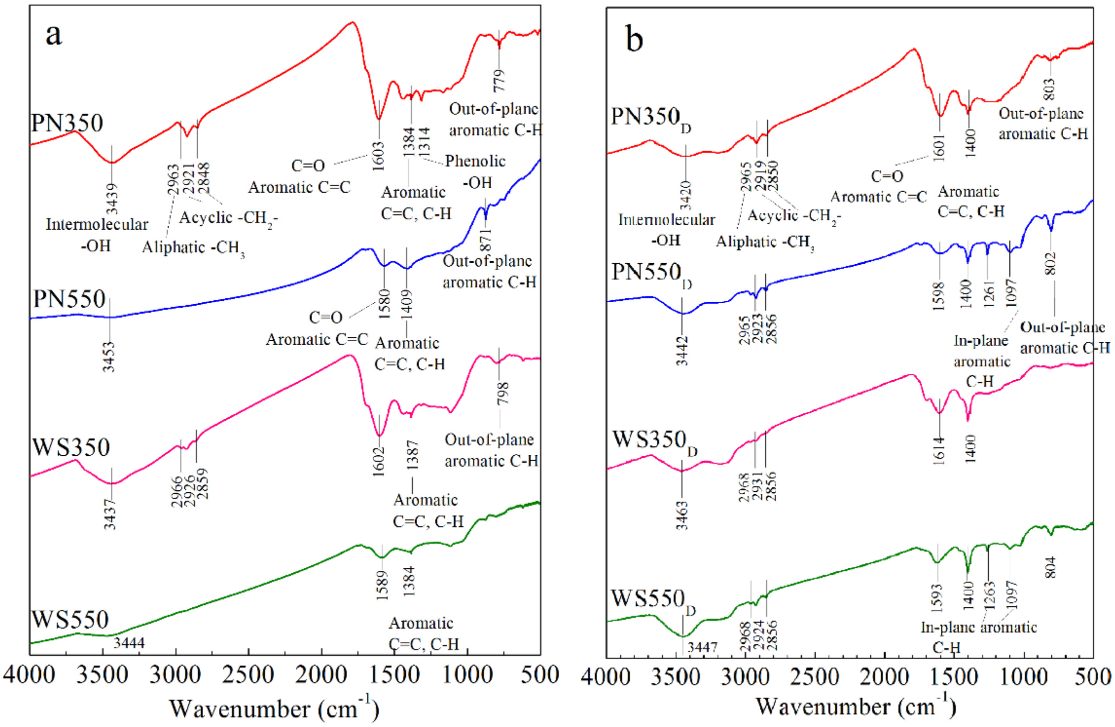

Figure S1 FTIR spectra of a) raw biochars and b) de-ashed biochars

The characteristic peaks of functional groups of different biochars were assigned and marked on Figure S1. The functional groups of biochars which produced at the same temperature were roughly the same. With the temperature increased from 350 °C to 550 °C, there was a decrease in peak intensities of intermolecular –OH (3437~3453 cm^{-1}), aliphatic –CH_3 (2963~2966 cm^{-1}), acyclic –CH_2– (2921~2926 cm^{-1} and 2848~2959 cm^{-1})[23] (Figure S1a), which implied the higher degree of carbonization of biochars produced at 550 °C relative to biochars produced at 350 °C. As can be seen from Figure S1b, the peak intensities of aromatic C=C and C-H (1400 cm^{-1}) increased after being washed by HCl/HF solution. The peaks of aliphatic –CH_3 (2965~2968 cm^{-1}), acyclic –CH_2– (2919~2931 cm^{-1} and 2850~2956 cm^{-1}) and the peaks of aromatic C-H (in-plane deformation vibrations) (1097~1263 cm^{-1}) of $PN550_D$ and $WS550_D$ become apparent compared with PN550 and WS550. The loss of ash by acid washing may result in the changes of these functional groups. The peaks which belonged to phenolic –OH (1314 cm^{-1}) in PN350 disappeared in the spectrum of $PN350_D$. These suggested that some phenolic compounds in PN350 were prone to release from biochar to the solution.

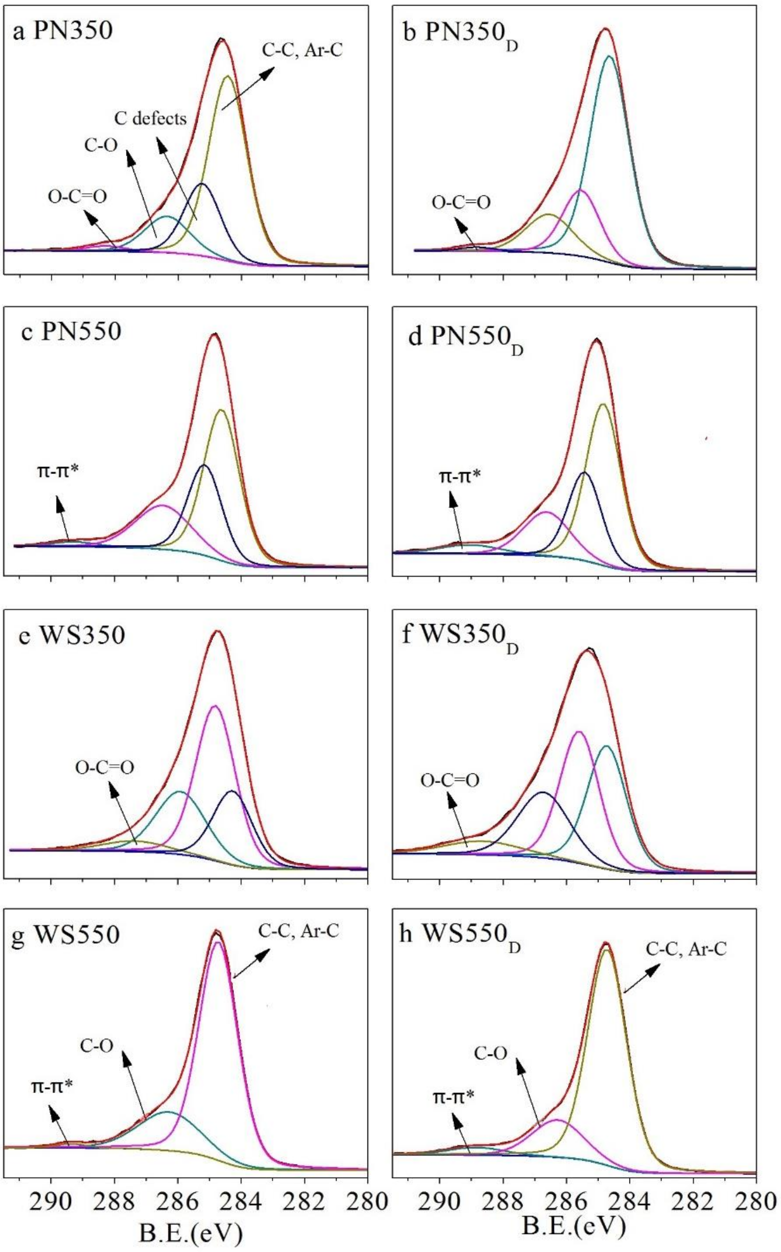

Figure S2 XPS C1s spectra of raw biochars (a, c, e, and g) and de-ashed biochars (b, d, f, h)

XPS has been considered as a useful method to characterize the functional groups on the surface of some carbon materials. To reveal the difference and transformation of the functional groups of the studied biochars, the XPS C1s spectra were analyzed. Deconvolution of C1s peaks of these biochars showed six types of carbon containing functional groups (Figure S2 and Table S1), i.e. C-C and Ar-C (aromatic carbon) (284.3~284.8 eV), C defects (carbon defects, 284.8~285.6 eV), C-O (285.9~286.7 eV),

C=O (287.5 eV), O-C=O (288.3~288.8 eV), and π-π*(288.8~289.4 eV).[24] As shown in Figure S2 and Table S1, C-C, carbon defects, and C-O were the main functional groups in PN350, PN550, and WS350. The functional groups of O-C=O and C=O which were respectively presented in PN350 and WS350 disappeared in PN550 and WS550, instead π-π* transition peak appeared in PN550 and WS550. This implied the increase of aromaticity in biochar structure with the increase of heating temperature. Compared with the fitting peaks of WS350, the carbon defect peak of WS550 seemed to be covered by the C-C peak, which may be caused by the low content of carbon defects in WS550. This suggested the higher degree of condensation of WS550 than that of WS350. The PPA of O-C=O of $PN350_D$ was much lower than that of PN350, this may be caused by the release of some organic matter during the acid washing treatment. The loss of C=O peak in WS350 C1s spectrum and the presence of O-C=O peak in $WS350_D$ C1s spectrum suggested the oxidation of functional groups occurred during acid washing. On the contrary, acid washing had little effect on the functional groups of biochar produced at 550 ^oC, which indicated that biochar prepared at high temperature are more resistant to oxidation.

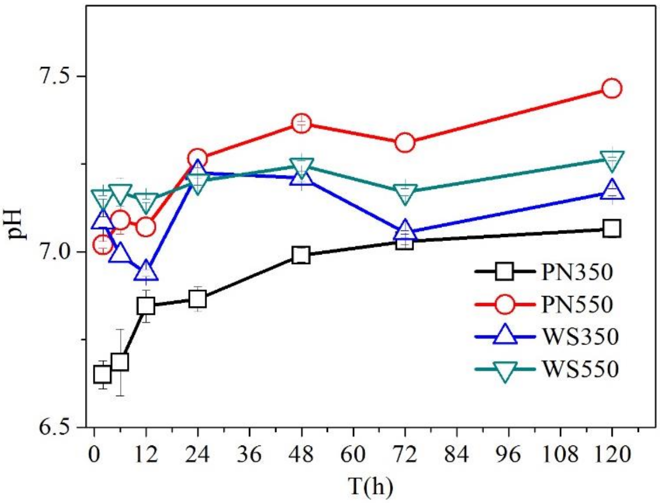

Figure S3 pHs of the mixtures of raw biochars and Zn solutions in kinetics experiment. Error bars represent ± SE (SE is the standard error of estimate). The experiments were conducted in duplicate (n = 2).

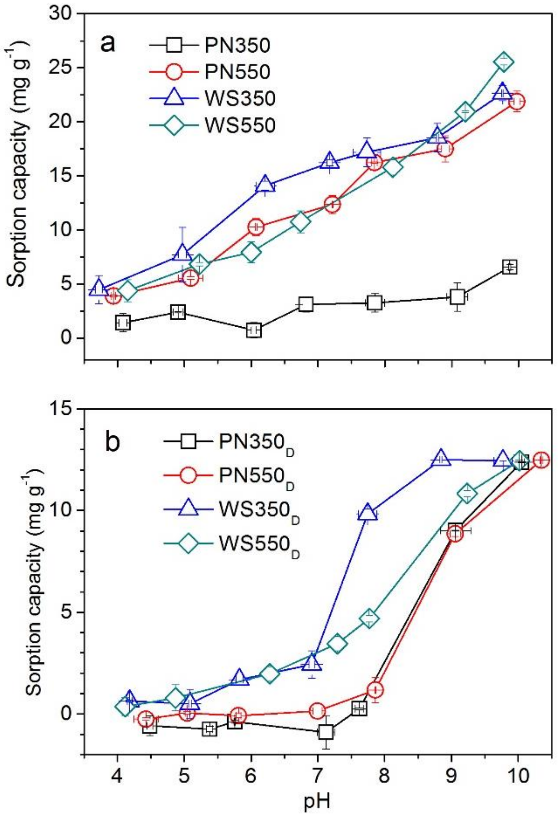

Figure S4 The effect of pH on sorption capacities of Zn on a) raw biochars and b) de-ashed biochars. The initial concentration of Zn was 150 mg L^{-1} for raw biochars, and 50 mg L^{-1} for de-ashed biochars. Error bars represent ± SE (SE is the standard error of estimate). The experiments were conducted in duplicate (n = 2).

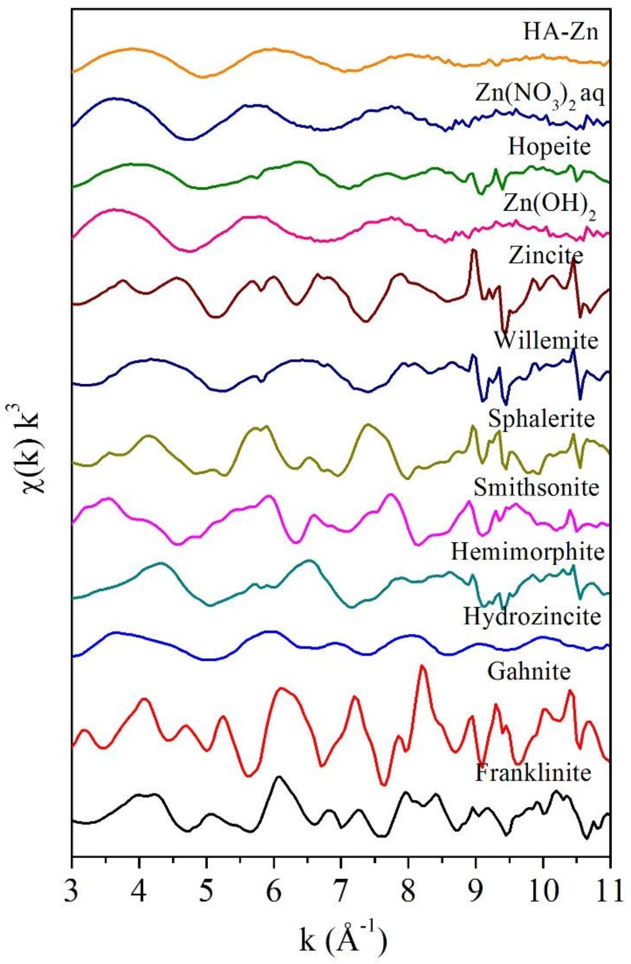

Figure S5 χ(k) k^3-spectra of standard Zn species

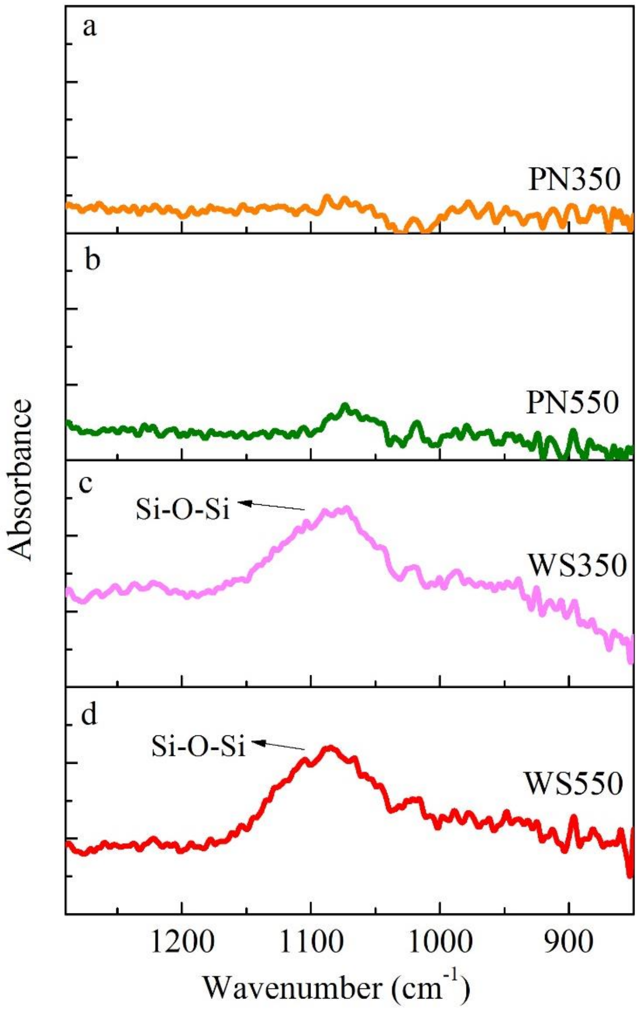

Figure S6 ATR-FTIR spectra of the filtrates of a) PN350, b) PN550, c) WS350, and d) WS550

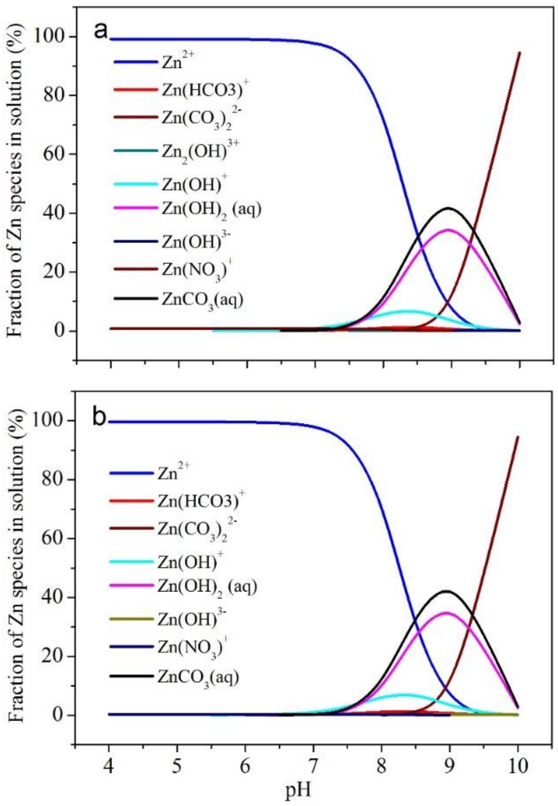

Figure S7 The fractions of Zn species in the solution when the initial Zn concentration was a) 150 mg L^{-1} and b) 50 mg L^{-1}

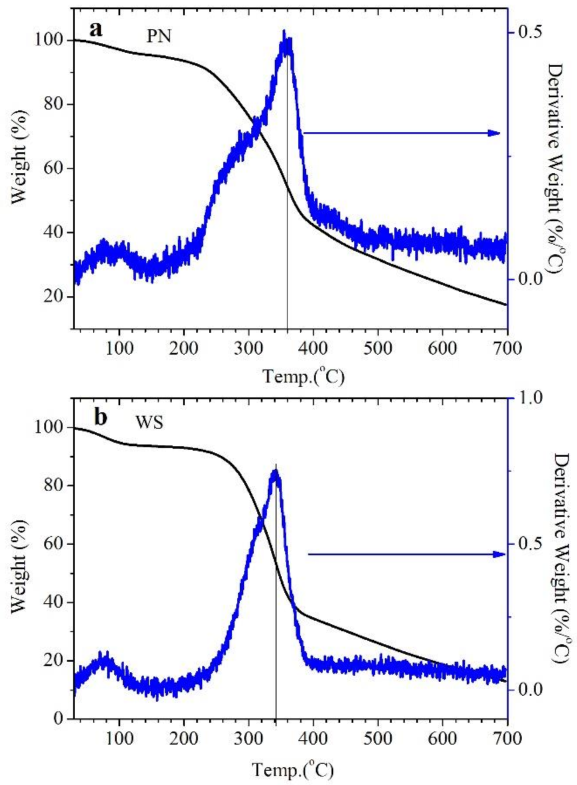

Figure S8 TG and DTG curves of a) PN and b) WS

REFERENCES

1. Scheinost, A. C., Kretzschmar, R., Pfister, S. & Roberts, D. R. Combining selective sequential extractions, X-ray absorption spectroscopy, and principal component analysis for quantitative zinc speciation in soil. *Environ. Sci. Technol.* **36,** 5021-5028 (2002).

2. Ressler, T., Wong, J., Roos, J. & Smith, I. L. Quantitative speciation of Mn-bearing particulates emitted from autos burning (methylcyclopentadienyl)manganese tricarbonyl-added gasolines using XANES Spectroscopy. *Environ. Sci. Technol.* **34,** 950-958 (2000).

3. Isaure, M. P. et al. Quantitative Zn speciation in a contaminated dredged sediment by μ-PIXE, μ-SXRF, EXAFS spectroscopy and principal component analysis. *Geochim. Cosmochim. Ac.* **66,** 1549-1567 (2002).

4. Luo, Y., Giammar, D. E., Huhmann, B. L. & Catalano, J. G. Speciation of Selenium, Arsenic, and Zinc in Class C Fly Ash. *Energ. Fuel.* **25,** 2980-2987 (2011).

5. Sarret, G.et al. Zn speciation in the organic horizon of a contaminated soil by micro-X-ray fluorescence, micro- and powder-EXAFS spectroscopy, and isotopic dilution. *Environ. Sci. Technol.* **38,** 2792-2801 (2004).

6. Webb, S. SIXpack: a graphical user interface for XAS analysis using IFEFFIT. *Physica Scripta 2005,* 1011 (2005).

7. Kim, S. S., Kim, J., Park, Y. H. & Park, Y. K. Pyrolysis kinetics and decomposition characteristics of pine trees. *Bioresour. Technol.* **101,** 9797-9802 (2010).

8. Müller-Hagedorn, M., Bockhorn, H., Krebs, L. & Müller, U. A comparative kinetic

study on the pyrolysis of three different wood species. *J. Anal. Appl. Pyrol.* **68–69,** 231-249 (2003).

9. Yang, H., Yan, R., Chen, H., Lee, D. H. & Zheng, C. Characteristics of hemicellulose, cellulose and lignin pyrolysis. *Fuel* **86,** 1781-1788 (2007).

10. Chan, K. Y. & Xu, Z. H. Biochar: Nutrient properties and their enhancement. *In Biochar for Environmental Management: Science and Technology* (ed. Lehmann, J. & Joseph, S.) 67-81 (London, 2009).

11. Lee, J. W. et al. Characterization of biochars produced from cornstovers for soil amendment. *Environ. Sci. Technol.* **44,** 7970-7974 (2010).

12. Yuan, T., Tahmasebi, A. & Yu, J., Comparative study on pyrolysis of lignocellulosic and algal biomass using a thermogravimetric and a fixed-bed reactor. *Bioresour. Technol.* **175,** 333-341 (2015).

13. Ho, Y. S.& McKay, G. Pseudo-second order model for sorption processes. *Process Biochem.* **34,** 451-465 (1999).

14. Chien, S. H. & Clayton, W. R. Application of elovich equation to the kinetics of phosphate release and sorption in soils. *Soil Sci. Soc. Am. J.* **44,** 265-268 (1980).

15. Zhou, Y. T., Branford-White, C., Nie, H. L. & Zhu, L. M. Adsorption mechanism of Cu^{2+} from aqueous solution by chitosan-coated magnetic nanoparticles modified with α-ketoglutaric acid. *Colloid. Surface. B.* **74,** 244-252 (2009).

16. Tütem, E., Apak, R. & Ünal, Ç. F. Adsorptive removal of chlorophenols from water by bituminous shale. *Water Res.* **32,** 2315-2324 (1998).

17. Ho, Y. S., Chiu, W. T. & Wang, C. C., Regression analysis for the sorption

isotherms of basic dyes on sugarcane dust. *Bioresour. Technol.* **96,** 1285-1291 (2005).

18. Dastgheib, S. A., Karanfil, T. & Cheng, W., Tailoring activated carbons for enhanced removal of natural organic matter from natural waters. *Carbon* **42,** 547-557 (2004).

19. Khaokaew, S., Landrot, G., Chaney, R. L., Pandya, K. & Sparks, D. L., Speciation and release kinetics of zinc in contaminated paddy soils. *Environ. Sci. Technol.* **46,** 3957-3963 (2012).

20. Cai, X., Huang, Q. X., Alhadj-Mallah, M. M., Chi, Y. & Yan, J. H., Characterization of zinc vapor condensation in fly ash particles using synchrotron X-ray absorption spectroscopy. *J. Zhejiang Univ-SC. A* **16,** 70-80 (2015).

21. Weber, W. J. & Morris, J. C. Kinetics of adsorption on carbon from solution. *Journal of the Sanitary Engineering Division* **89,** 31-60 (1963).

22. Foo, K. Y. & Hameed, B. H. Insights into the modeling of adsorption isotherm systems. *Chem. Eng. J.* **156,** 2-10 (2010).

23. Uchimiya, M., Wartelle, L. H., Klasson, K. T., Fortier, C. A. & Lima, I. M. Influence of pyrolysis temperature on biochar property and function as a heavy metal sorbent in soil. *J. Agr. Food Chem.* **59,** 2501-2510 (2011).

24. Datsyuk, V. et al. Chemical oxidation of multiwalled carbon nanotubes. *Carbon* **46,** 833-840 (2008).
